# Enhanced optical efficiency and color purity for organic light-emitting diodes by finely optimizing parameters of nanoscale low-refractive index grid

**DOI:** 10.1038/s41598-020-62470-5

**Published:** 2020-03-27

**Authors:** Jae Geun Kim, Yooji Hwang, Ha Hwang, Jun Hee Choi, Young Wook Park, Byeong-Kwon Ju

**Affiliations:** 1 Display and Nanosystem Laboratory, School of Electrical Engineering, Korea University, Seoul, 02841 Republic of Korea; 2 The School of Mechanical and ICT Convergence Engineering, SUN MOON University, Chungcheongnam-do, 31460 Republic of Korea

**Keywords:** Electronic devices, Organic LEDs

## Abstract

To extract the confined waveguided light in organic light-emitting diodes (OLEDs), inserting a low refractive index (RI) periodic structure between the anode and organic layer has been widely investigated as a promising technology. However, the periodic-structure-based light extraction applied inside devices has been shown to severely distort spectrum and affect EL characteristics. In this study, a simple light extraction technology using periodic low-RI nanodot array (NDA) as internal light extraction layer has been demonstrated. The NDA was fabricated simply via laser interference lithography (LIL). The structural parameters of periodic pattern, distance, and height were easily controlled by the LIL process. From computational analysis using finite-difference time-domain (FDTD) method, the NDA with 300 nm pitch and 0.3 coverage ratio per unit cell with 60 nm height showed the highest enhancement with spectral-distortion-minimized characteristics. Through both computational and experimental systematic analysis on the structural parameters of low-RI NDA-embedded OLEDs, highly efficient OLEDs have been fabricated. Finally, as representative indicators, hexagonal and rectangular positioned NDA-embedded OLEDs showed highly improved external quantum efficiencies of 2.44 (+29.55%) and 2.77 (+57.38%), respectively. Furthermore, the disadvantage originating from the nanoscale surface roughness on the transparent conductive oxide was minimized.

## Introduction

Screen, or display, has been acting as a connector between human being and electronic device for many decades. Because the need for advanced display by consumers and the market has been increasing, many types of display for human consumption have become available in recent years.

Organic light-emitting diodes (OLEDs) are in the limelight because of their many advantages, such as natural color gamut, low consumption, fast response, and available adaptability for flexible devices. As the need for highly efficient OLEDs arises, numerous research papers are being published regarding the enhancement of these devices^1–6^. One of the researches to improve OLEDs is regarding light out-coupling because OLEDs intrinsically have light loss mechanisms, which include waveguide mode, substrate mode, and surface plasmon polariton (SPP) mode^7–11^. Basically, the quantity of light that comes out from the emissive layer is only under 20% because of the aforementioned loss reasons^12,13^. To overcome these problems, many ideas have been presented, including modifying the interface of layer to scatter confined light, inserting random structures or particles, and attaching lens arrays^14–22^.

Compared to the rest of these ideas, inserting nanoscale periodic structures composed of low-refractive-index material in OLEDs has advantages, such as high efficiency and increased color purity matched with the emission wavelength of OLEDs^23–26^. For example, one of the researches on PC OLEDs reported an over two-fold enhancement^27^.

However, most of these OLEDs equipped with periodic structure for light extraction have a distorted spectrum with a wide range of viewing angles^28,29^. On the other hand, the microscale periodic structure does not suffer from spectral distortion, but has a lower enhancement; Y. Sun *et al*. reported a ~32% enhancement in external quantum efficiency (EQE) with a 7 μm pitch microscale periodic structure^30^, while T-W Koh *et al*. reported a ~20% enhancement in EQE with a 6 μm pitch microscale periodic structure^31^. Furthermore, light extraction techniques that are independent of pixel size as the pixel size of the current display is getting smaller.

In this study, we propose a light outcoupling technology that uses low refractive index (RI) periodic nanosized dot arrays (NDA). Through systematic analysis, a highly efficient OLED has been demonstrated. The low RI NDA light extraction layer successfully extracted the wave-guided light efficiently by adopting the nanoscale period structure in low RI light extraction technology. Furthermore, the photonic crystal effect induced spectrum distortion which is commonly exhibited by periodic-structure-based devices was minimized by controlling the structural parameters of periodic RI NDA: pitch, height, and arrangement. The exhibited low RI periodic NDA light extraction layer has been fabricated simply by using laser interference lithography (LIL). Finally, under the same conditions for low RI NDA, OLEDs equipped with low RI NDA light extraction layers with hexagonal and rectangular arrangements exhibited +29.55% and +57.38% enhanced EQEs, respectively. The OLEDs with hexagonal arrangement also showed lower efficiency enhancement but minimized spectral distortion and increased color purity better than the OLEDs with rectangular arrangement. Furthermore, the electrical short, which has been easily found in surface roughness induced by fabricating the NDA directly on the top surface of bottom electrode through light extraction technology, was not observed here. The results were fundamentally analyzed using the finite-difference time-domain (FDTD) computational method.

## Results and discussion

### Fabrication of low-index NDA

**Figure 1:**
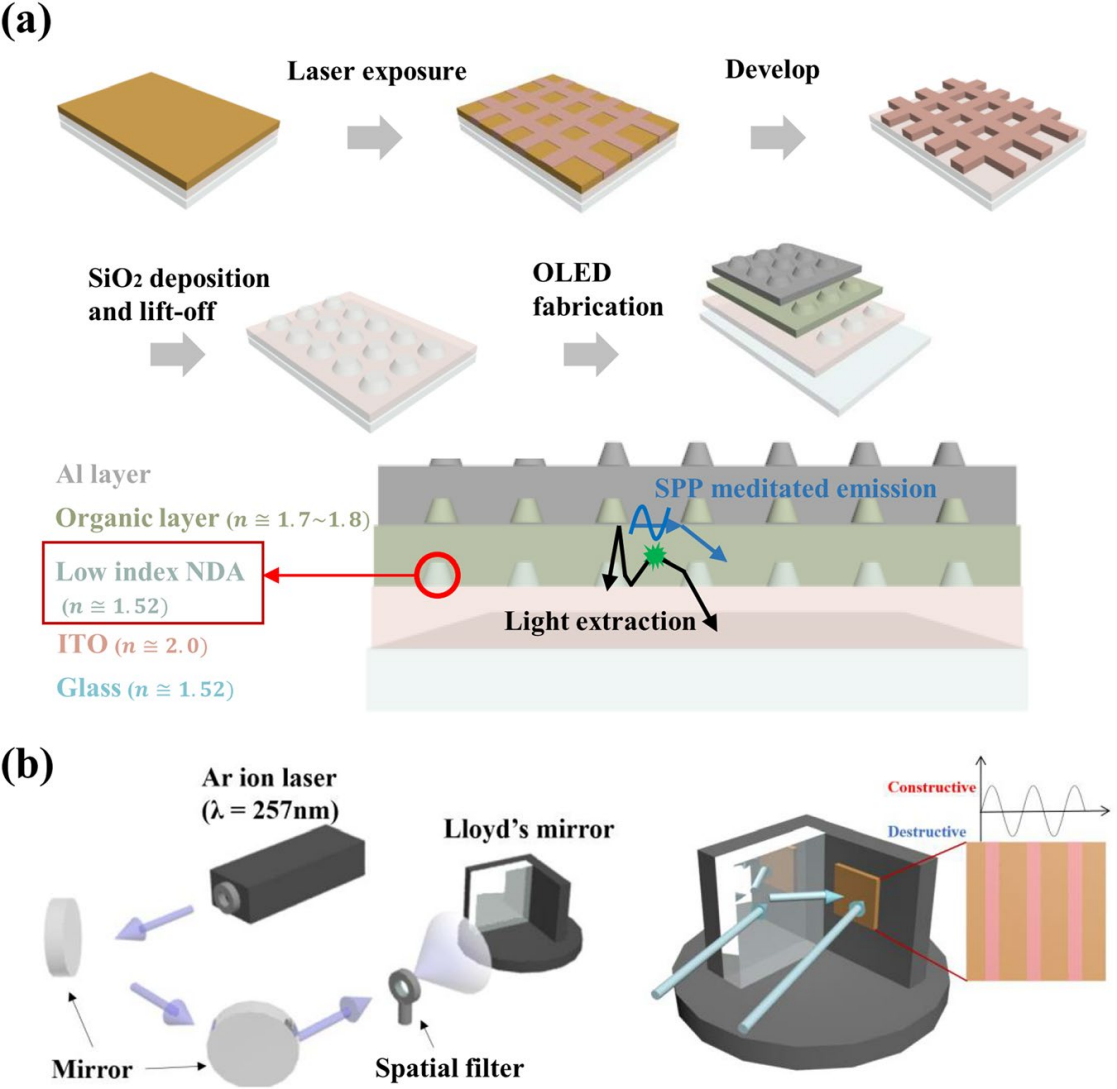
(**a**) Schematic diagram of fabrication process and (**b**) description of laser interference lithography.

In Fig. 1, the fabrication process for NDA-integrated OLEDs is presented. ITO coated glass was used as substrate and cleaned for spin-coating photoresist. Hexamethyldisilazane was spin-coated and baked for 3 min at 200 °C as an adhesion promoter for photoresist. The negative photoresist with thinner then spin-coated and baked for 1 min at 100 °C. For the LIL process to nanoscale patterning, 2-beam and 3-beam Lloyd’s mirror interferometer were used. When fabricating rectangular array, the PR-coated sample was twice exposed to a Ar-ion continuous wave laser with 2-beams Lloyd’s mirror. Hexagonal arrayed sample was fabricated with once exposure to Ar-ion continuous wave laser with 3-beams Lloyd’s mirror as described in Fig. S3. After exposure process, the development process was then followed. The hardened PR maintained a nanoscale rectangular or hexagonal arrangement depending on the usage of 2-beam or 3-beam Lloyd’s mirror. Using these grid patterns as a lift-off mask, the low-index NDA of silicone dioxide (SiO_2_, refractive index of 1.5), which was deposited by e-beam evaporator, was fabricated using a lift-off process. With regard to the unnecessary parts, the nanoscale grid PR mask was removed via sonication in acetone. Lastly, after the PR mask removal process, the low-index SiO_2_ NDA light extraction structure was demonstrated.

The periodic distance between each dot is defined as pitch, which is defined as below.

$${\Lambda }_{2-{beam}}=\lambda /(2\,\sin ({\rm{\theta }}))$$

$${\Lambda }_{3-{beam}}=3\lambda /(2\,\sin ({\rm{\theta }}))$$

Λ is the pitch of dot patterns, λ is the wavelength of laser, and θ is the angle between the directly incident wave and reflected wave from the mirror^32^.

**Figure 2:**
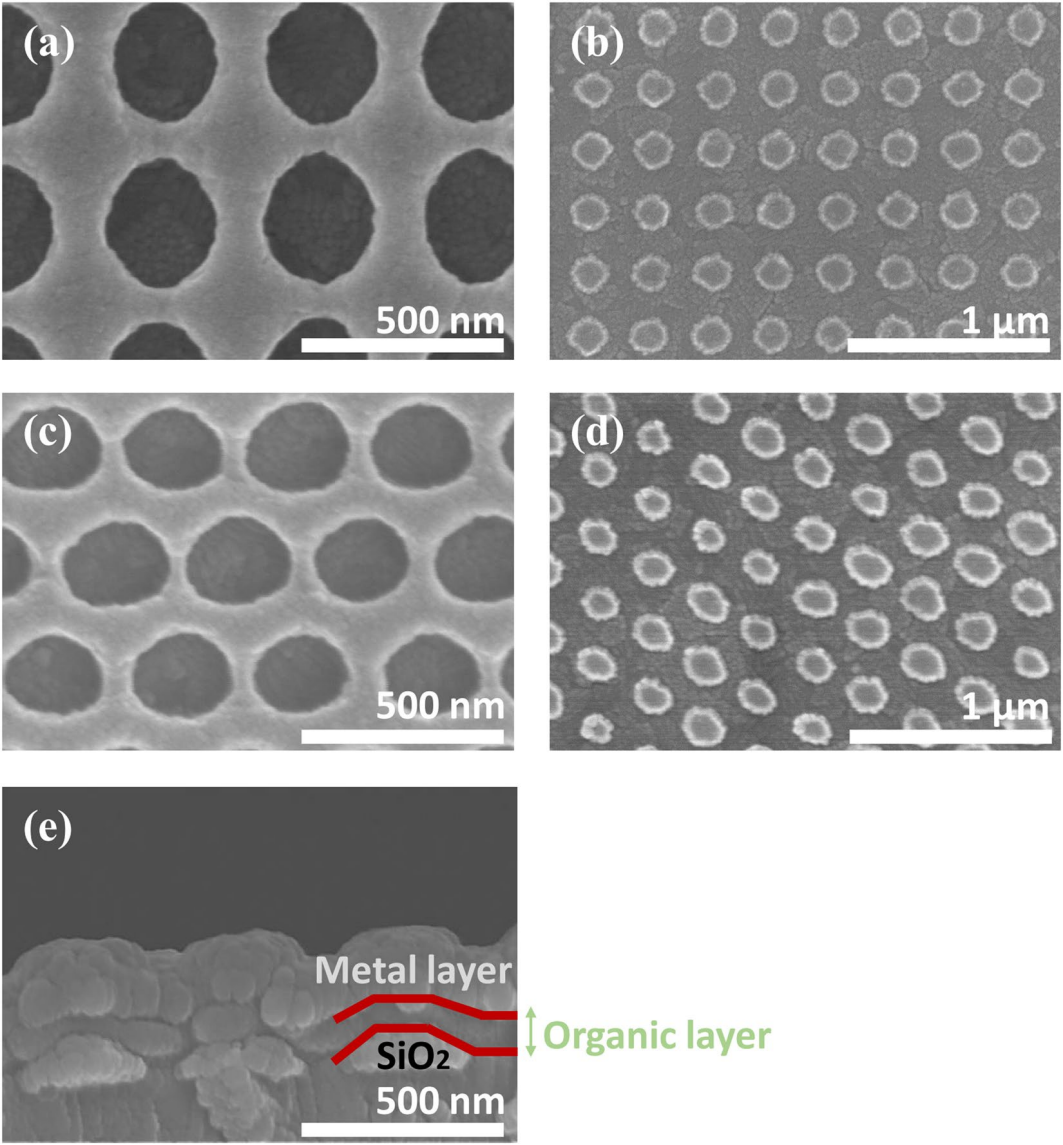
Fabricated dot pattern shapes composed of SiO_2_ low-index layer. (**a,c**) are patterned photoresist samples after LIL process. (**b,d**) are rectangular and hexagonal NDA arrays after lift-off process. (**e**) shows cross section of fabricated OLED device, with corrugated structure following the same pattern as that of the low-index NDL structure.

In Fig. 2, the SEM images of each fabrication step show how well the patterns and OLEDs are fabricated. Figure 2(a) shows the nanoscale-grid-patterned PR lift-off mask using LIL, Fig. 2(b,c) shows the rectangular–hexagonal SiO_2_ NDA, and Fig. 2(d) shows the cross-sectional image of the fabricated OLEDs. The NPB/Alq_3_/LiF/Al layers clearly follow the corrugated structure of the NDA. The observed fill factor, which means the occupying area per unit cell of NDA, was about 0.3, i.e., a single dot area occupies 30% per unit cell. Meanwhile, the tapered angle of the dot structure was approximately 45°.

Low-index NDA integrated OLEDs have been successfully devised using this simple LIL process, and are expected to have enhanced EL characteristics.

In our device, the following mechanisms are mainly expected to result in enhancing efficiencies via the orientation of the nanoscale corrugated structure. (1) Extracting waveguide-mode light by modifying the ray’s propagation using the refractive index difference of the inserted NDA between the ITO and adjacent organic layer, and by using an efficient taper angle of the NDA structure^30,31^. (2) Extracting trapped surface plasmon polariton (SPP) mode that originated from resonance^33^.

Although the advantages of light extraction through the introduction of low RI NDA have been previously described, the fabricated OLED devices have a clear trade-off relationship. Simply considering that the covered NDA structure of SiO_2_ acts like an electrical insulator, the current density of low-index NDA integrated OLEDs decreases with respect to the reference at the same applied voltage, as shown in Fig. S4(a). In other words, according to the aforementioned perspective, the emitting area of emission layer (EML) is decreased. Thus, if the single dot pattern is sufficiently optimized, the reduction of the emitting area can be complemented by the light extraction effect^30,31^.

The NDA shape parameter (height, pitch) induced photonic enhancement and the NDA coverage area rate should therefore be carefully considered.

### Optimization of NDA parameters via FDTD simulation

The NDA parameter (pitch, height) dependent light extraction enhancement was carefully calculated using finite differential time domain (FDTD) methods for optical analysis. The overall FDTD simulation was conducted in two steps. First, we optimized the optimal point of pitch and height of NDA through 2-dimensional simulation. We then profoundly analyzed the spectrum and viewing angle characteristics by distinguishing the arrangement of NDA through 3-dimensional simulation.

**Figure 3:**
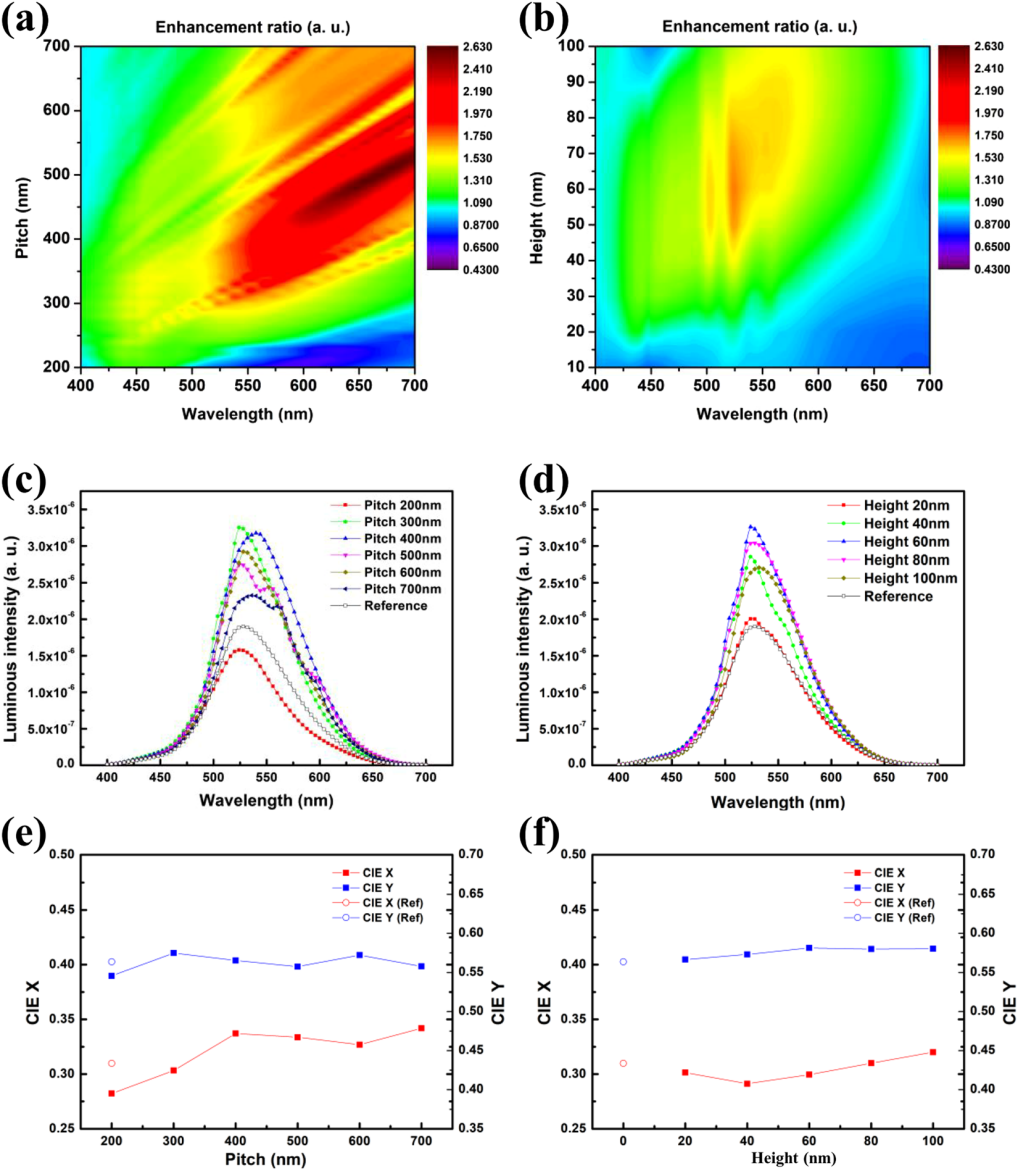
2-Dimensional FDTD calculation results of light extraction enhancement using NDA parameter with line arrangement. (**a**) Varied pitch with 60 nm height and 0.3 fill factor, and (**b**) varied height with 300 nm pitch and 0.3 fill factor. (**c**) Luminous intensity varied pitch with 60 nm height and 0.3 fill factor, and (**d**) varied height with 300 nm pitch and 0.3 fill factor. Variation in CIE with changes in (**e**) pitch and (**f**) height of NDA.

Figure 3(a) shows the results on how the enhancement factor changes with pitch. The characteristics of the photonic crystal are clearly shown by the dependence on the Bragg’s diffraction condition. As evidence of Bragg’s first-order diffraction, the highest enhancement occurs at the first order; the comparison was done with respect to other orders of diffraction at specific wavelengths matching the emission wavelength range. Furthermore, in the case of Bragg’s second-order diffraction, it shows a more weakened tendency to extract light at certain wavelengths based on photonic crystal theory. To develop a highly efficient green light emitting device, the pitch has been chosen to be 300 nm.

Simulation results of the expected OLED device when the height of the NDA was changed are illustrated in Fig. 3(b), with the pitch of the NDA fixed at 300 nm. When the height of the NDA was more than 20 nm, the efficiency was improved more than that of a flat device without NDA structure. This means that the light, which is guided mainly in the waveguide mode, reaches the NDA structure, and a light-extracting effect is demonstrated when a sufficient height for the NDA is established. Furthermore, full-scale spectrum distortion and high-efficiency improvement occurred when the NDA height was between 40 nm and 80 nm.

Figure 3(c,d) show the relative luminous intensity spectra varying with pitch and height of NDA, which are from Fig. 3(a,b), respectively.

In Fig. 3(c), simulation results for a pitch of 200 nm show little spectral distortion but a low efficiency enhancement relative to the reference, which does not include the NDA layer. On the other hand, simulation results for above 300 nm of pitch show improved efficiency relative to the reference and shifted peak point of spectrum varying with pitch. Among the results, 300 nm of pitch show appropriately high efficiency and lowest shifted peak point (528 nm to 526 nm, Δ2 nm) of spectrum.

The expected luminous spectra according to the height change of the NDA are shown in Fig. 3(d), with pitch of the NDA fixed at 300 nm. The expected increase in efficiency, for NDA heights ranging from 20 nm to 100 nm, was best when the NDA height was 60 nm.

Figure 3(e,f) illustrate the variation of CIE X and Y when each parameter, i.e., pitch and height, of NDA changes. As previously mentioned, in order to minimize the change of CIE while maintaining the condition of high efficiency, we chose 300 nm pitch and 60 nm height as the optimized condition for NDA.

**Figure 4:**
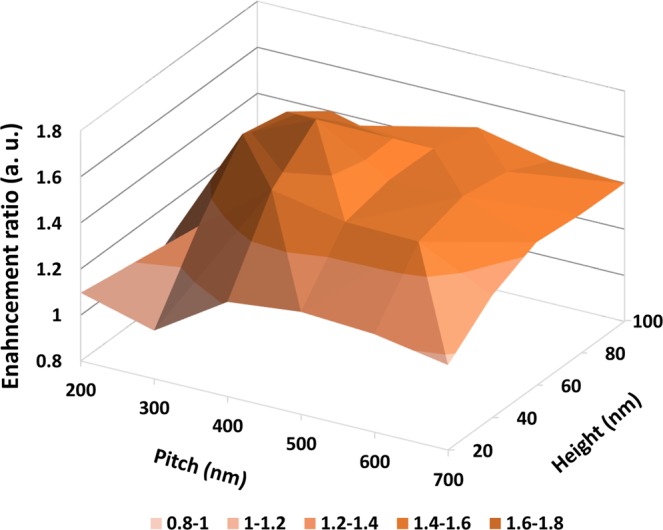
2-Dimensional FDTD calculation results on enhancement ratio as a function of pitch and height, with respect to whether NDA is adapted or not.

Figure 4 clearly shows the enhancement ratio when the pitch and height are used as functions for NDA optimization. The enhancement ratio was calculated by summing the relative luminous intensities in the full width at half minimum (FWHM) emission wavelength region and dividing the sum by the relative luminous intensity of the reference, which does not include an NDA layer. As mentioned previously on Fig. 3(c), we selected the second highest efficiency pitch 300 nm and height 60 nm case as the best point for the NDA, except when the NDA pitch and height are 400 nm and 60 nm, respectively, which show the highest efficiency but with a severe shifted peak point of emission spectrum (528 nm to 540 nm, Δ12 nm). For a more profound understanding of the wavelength enhanced by the NDA layer, Fig. S1 shows the luminous intensity, as detected by the monitor during the FDTD simulation, according to the change of pitch and height. The relative luminous intensity with respect to the emission angle clearly shows that the enhanced light in the wavelength range according to each transverse electric (TE), transverse magnetic (TM) mode is clearly visible. When the pitch and height of NDA are 300 nm and 60 nm, respectively, the enhanced wavelength almost coincides with the peak wavelength of Alq3, which is the actual emitting layer of the fabricated OLED device. Furthermore, simulation results including the NDA layer show, via center-concentrated emission, that the intensity of outcoupled light with the NDA layer is higher at a low emission viewing angle under 30°, compared to those of other ranges. The enhancement ratio of FDTD calculation results are summarized in Table 1 varying with pitch and height.

**Table 1:** Summary of FDTD calculation results on enhancement ratio.

		Height				
		20 nm	40 nm	60 nm	80 nm	100 nm
Pitch	200 nm	1.10	0.91	0.80	0.80	0.89
	300 nm	1.01	1.32	1.58	0.80	0.80
	400 nm	1.21	1.56	1.72	1.56	1.30
	500 nm	1.12	1.35	1.48	1.38	1.34
	600 nm	1.25	1.52	1.49	1.57	1.53
	700 nm	1.17	1.33	1.33	1.40	1.40

**Figure 5:**
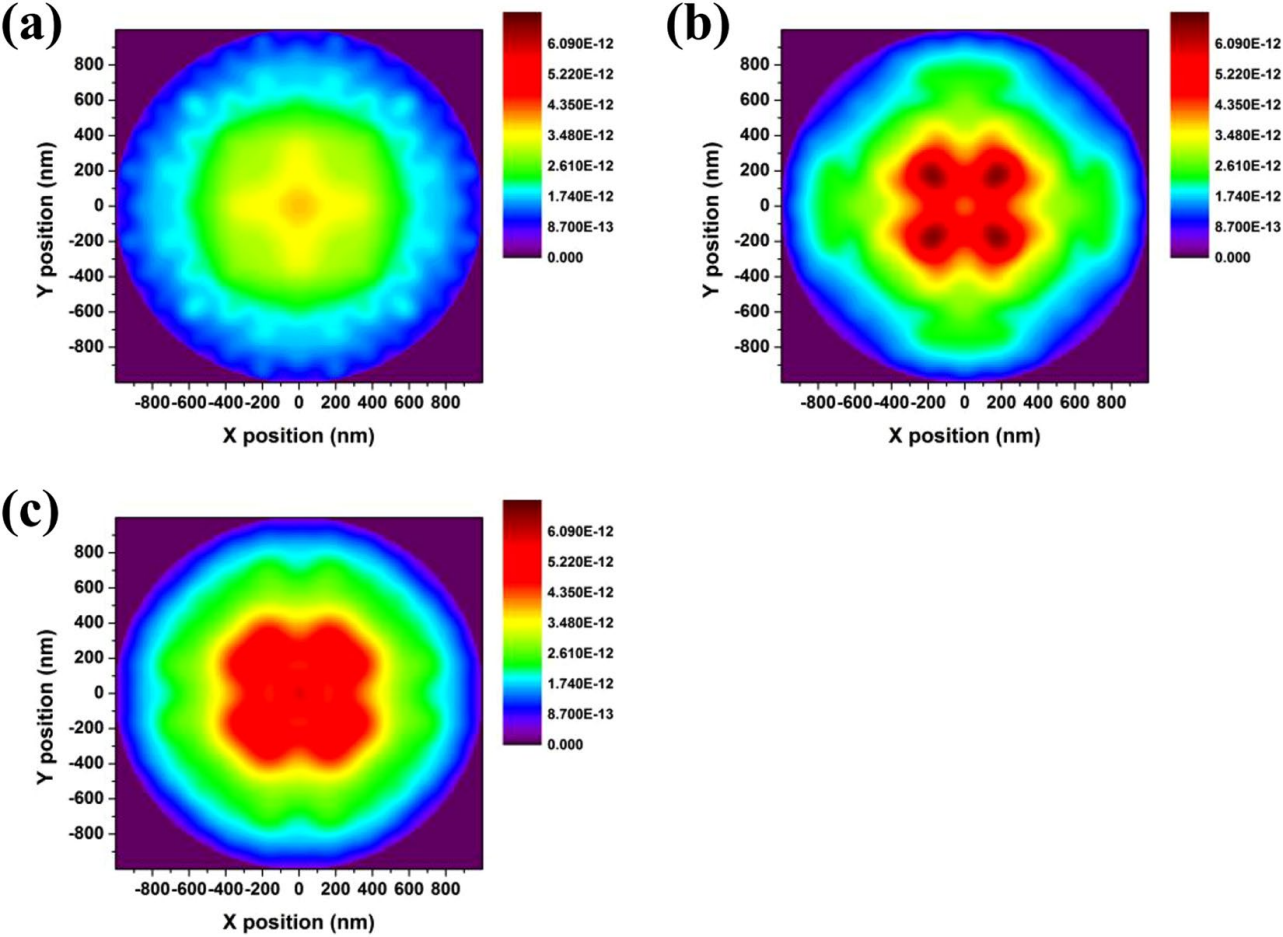
3-Dimensional FDTD calculation results on relative luminous intensity with respect to viewing angle. These results were calculated under the assumption that an interface exists in the far field. (**a**) Calculated results of the reference, which does not have NDA layer. NDA layer inserted cases with (**b**) rectangular array and (**c**) hexagonal array, respectively.

Relative electric field intensities from 3-dimensional FDTD simulation results according to arrays are presented in Fig. 5. In Fig. 5(a), the relative electric field of the flat reference model without NDA shows a smooth distribution that is similar to a Lambertian distribution. On the other hand, as light outcoupling occurs in the direction perpendicular to the horizontal device surface because of the periodic NDA nanostructure, the models with the NDAs characterized by rectangular and hexagonal arrays have the highest intensities at 10° and 40°, respectively, as shown in Fig. 5(b,c), respectively. The relative electric field intensities are shown to be relatively smoother for the hexagonal model than for the rectangular model. This is because in the model with the rectangular array, the adjacent pattern is relatively distant and the range in which the adjacent pattern exists according to the scattering direction is narrow, making it difficult for secondary scattering to occur. In the model with the hexagonal array, secondary scattering can contribute largely to the result; this is similar to a scattering effect due to random patterns, and the distortion appears to be reduced.

### Characteristics of OLED devices equipped with NDA layer

After the parameters of the NDA were optimized, green fluorescence OLED devices with rectangular (Device-1) and hexagonal (Device-2) arrays were fabricated. The reference (Ref) device structure was Glass/ITO (180 nm)/NPB (60 nm)/Alq_3_ (80 nm)/LiF (0.7 nm)/Al (100 nm). The device structures of Device-1 and Device-2 were the same as that of Ref, except that NDA existed between the ITO and NPB layers.

**Figure 6:**
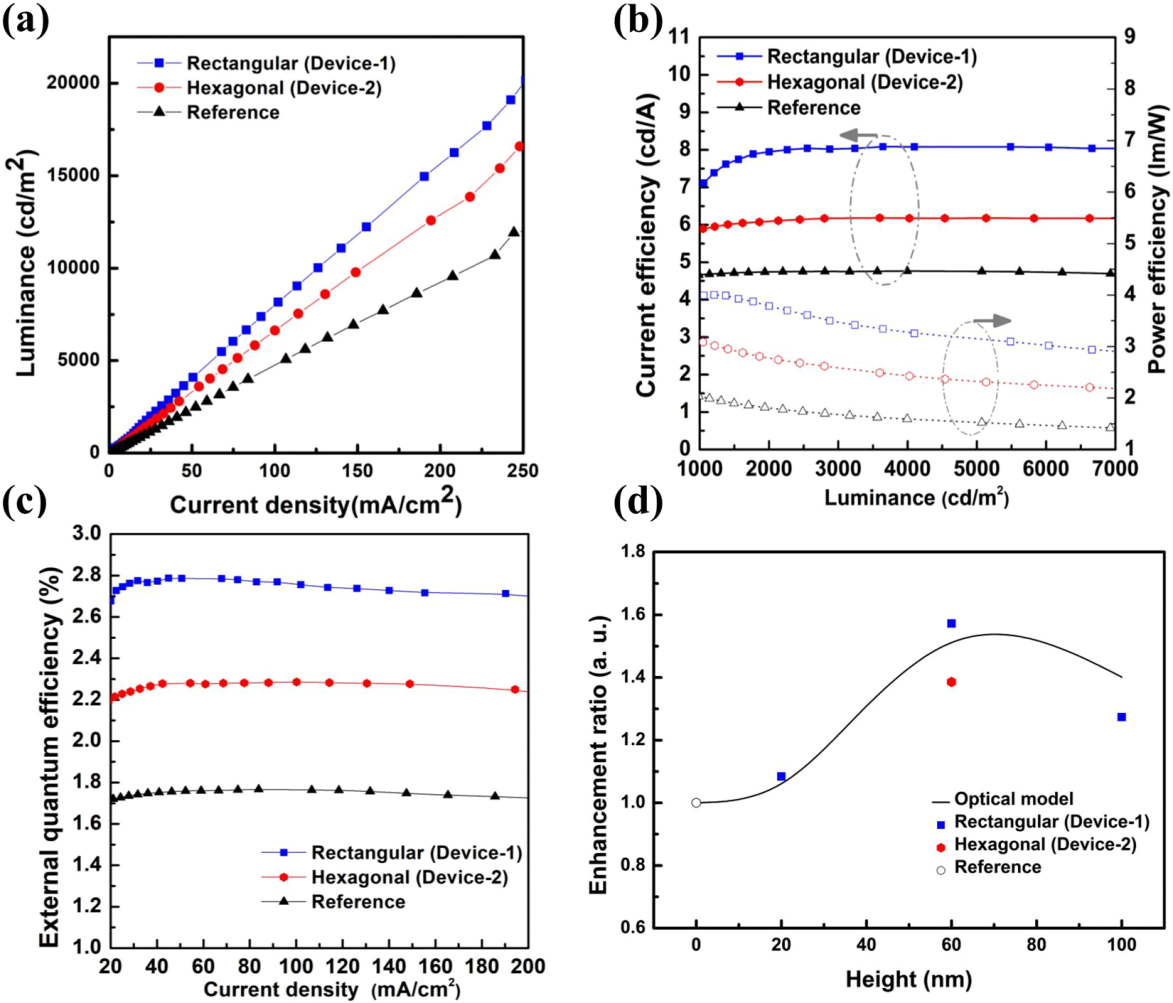
EL characteristics of fabricated OLEDs. All the fabricated OLED devices have the same NDA structure (pitch and height are 300 nm and 60 nm). (**a**) Luminance versus current density. (**b**)The current efficiency and power efficiency versus luminance. (**c**) External quantum efficiency versus current density. (**d**) Relative enhancement ratio of OLED devices compared to reference OLED device versus height of NDA.

Both Device-1 and Device-2 showed higher luminance at the same current density than the Ref device due to the light outcoupling effect of the optimized NDA structure in Fig. 6(a). As summarized in Table 2, the current efficiencies and power efficiencies were greatly enhanced to 8.02 (+68.49%) and 6.61 (+30.32%), respectively, for Device-1, and 3.5 (+105.88%) and 2.81 (+50.00%), respectively, for Device-2, both at 3000 cd/m^2^. The current efficiencies and power efficiencies showed constant enhancement ratios relative to Ref until the luminance of Ref reached 12,000 cd/m^2^, the maximum value in Fig. 6(b). It means that stable improved efficiencies occurred at all ranges that we measured. Therefore, as shown in Fig. 6(c), the EQEs of Device-1 and Device-2 were also increased to 2.77 (+57.38%) and 2.44 (+29.55%), respectively, with respect to those of Ref.

**Table 2:** Summary of EL characteristics of fabricated OLEDs at 3000 cd/m^2^.

	Current efficiency [cd/A]	Power efficiency [lm/W]	External quantum efficiency [%]
Device-1	8.02 (+68.49%)	3.50(+105.88%)	2.77(+57.38%)
Device-2	6.19(+30.32%)	2.50(+50.00%)	2.28(+29.55%)
Ref.	4.76	1.70	1.76

The mechanism that majorly enhances these devices is their capability to significantly extract trapped waveguide mode and surface plasmon polariton mode. In waveguide mode, the inserted NDA structure modifies the wave-guided ray’s propagation at the interface between NDA and NPB layers via the dependence to the critical angle by the refractive index. Furthermore, NDA leads these trapped ray’s modes extracted normally to the interface, resulting in lights from NDA devices having a greater directional property than those of the Ref device. Because of this reason, devices with NDA heights over 60 nm represent specific enhancements due to 2D periodic grating and demonstrate higher efficiencies than expected.

$${{\rm{k}}}_{0}\,\sin \,{\rm{\theta }}=\pm {{\rm{k}}}_{{\rm{wg}}}\pm {{\rm{k}}}_{{\rm{g}}}=\pm \frac{2{\rm{\pi }}{\rm{n}}}{{\rm{\lambda }}}\pm {\rm{m}}\frac{2{\rm{\pi }}}{\Lambda }$$

Another reason behind the enhancement is that the fabricated devices have corrugated structures, which affect the extraction of trapped photons, SPP, caused by surface plasmon via breaking resonance. As we mentioned previously on Fig. 3, this corrugated structure makes $\overrightarrow{{{\rm{E}}}_{{\rm{TE}}}}\cdot \overrightarrow{{{\rm{E}}}_{{\rm{SP}}}}\ne 0$. Because of this dispersion relation, more trapped light can be coupled out.

Figure 6(d) shows the relationship between the height of the fabricated OLED devices and the height of the NDA when its pitch is 300 nm. The enhancement ratio increases until the height of the NDA reaches 60 nm as maximum point, and then decreases again. This tendency occurs in both the simulation results and the fabricated OLED devices.

**Figure 7:**
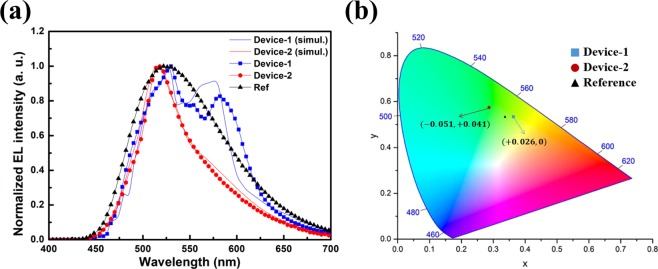
(**a**) The EL spectra of OLEDs with RI NDA. For comparison, the experimental results and FDTD simulation results are presented. (**b**) CIE color coordinate and change of Device-1, Device-2, and reference.

The spectra of fabricated OLED devices and corresponding FDTD simulation results are shown in Fig. 7(a). The spectra of the fabricated devices show a tendency to match satisfactorily, considering that the FDTD results were calculated under ideal conditions. Even if the fabricated OLED devices have the same structural parameters for periodic RI NDA, occurrence of multiple peak points in spectrum depends on their arrangement of NDA.

This is assumed to be influenced by the scattering effects from the order of the periodic low RI NDA that can induce light extraction, and by the density of NDA per unit cell.

Considering the first order of the scattering effect, for hexagonal array, the first distance between NDAs is identically 300 nm when the pitch is 300 nm. However, in the rectangular array case, the arrangement of NDAs with a distance of about 424 nm is present as a diagonal pitch. Specific enhancements originating from the diagonal pitch can also be observed in Fig. 3(a) from the FDTD simulation results.

From the viewpoint of the density of NDA, the hexagonal case is denser than the rectangular case. Therefore, in the hexagonal array case, scattering effect due to orders higher than the first order of NDA occurs more significantly than in the rectangular case and leads to the enhancement peak point being strengthened without another peak point.

As a result of the aforementioned phenomenon, Device-2 has a higher color purity because its wavelength band, from the full width at half maximum (FWHM) in Fig. 7(a), is clearly reduced (494 nm to 558 nm, Δ64 nm) compared to that of Reference (480 nm to 600 nm, Δ120 nm).

In Fig. 7(b), the CIE variations of the fabricated OLED devices are shown. The CIE coordinate of Device-1 is shifted to the reddish region because the secondary enhanced peak is near the 580 nm wavelength. In Device-2, as described previously, CIE changes in the direction of increasing color purity as the intensity of spectrum decreases in the long wavelength region based on the peak point of the reference, under the condition that the parameters of the NDA were optimized for minimizing the distortion of spectra.

In conclusion, from the computational analysis and the experimental results, it was found that the characteristics of OLED devices change according to the fine parameter change of the low RI NDA. First, a profound FDTD simulation analysis was performed to determine the optimized RI NDA conditions (pitch 300 nm, height 60 nm) that minimized spectral distortion but maximized efficiency improvement. RI NDAs with the aforementioned optimized conditions were fabricated simply into hexagonal and rectangular arrangements through the LIL process. The external quantum efficiencies of OLED devices having hexagonal and rectangular arrangements of RI NDA were enhanced by +29.55% and 57.38%, respectively. From the perspective of efficiency, that of the OLED device with rectangular arrangement of RI NDA was higher than that of the OLED device with hexagonal arrangement of RI NDA; however, the color purity and degree of minimized spectrum distortion exhibited opposite trends. Similar approaches previously reported on the improvement of OLEDs characteristics by introducing periodic structure into OLEDs are also summarized in Table S2 to compare with our study.

It is promising that the described fabrication process is a simple, fast, and large-area-adaptive process, which is advantageous for commercialization. Furthermore, because the RI NDA structure has a separate dot structure, it is hopeful that it can be applied to light extraction of flexible OLED devices in the future.

## Methods

### Low-index NDA

The low-index NDA layer is composed of SiO2 which has a large refractive index difference compared to organic layers. Fabrication process of low-index NDA layer is as follows. Glass coated with 185 nm thickness indium tin oxide (ITO) with sheet resistance of 10 Ω/sq was used as substrate (Samsung Corning Co., Ltd.). The substrate was finely cleaned via ultrasonication with acetone, methanol, and deionized water, in that order. Hexamethyldisilazane (Sigma Aldrich Co., Ltd.) was spin-coated as an adhesion promoter between negative photoresist (PR, AR-N 4240, Allresist GmbH) and ITO, followed by baking on a hot plate for 3 min at 200 °C. The PR mixed with thinner (AR 300–12, Allresist GmbH) was spin-coated, and baked on a hot plate for 1 min at 100 °C. The PR-coated sample was exposed to a 257 nm Ar-ion continuous wave laser (Lexel 95 SHG, Cambridge Lasers Laboratories, Inc.) with an energy density of 10 mJ/cm^2^. After the exposure process of the PR-coated sample, development was performed by MIF-300 (AZ electronic Materials Co., Ltd.) with diluted deionized water for 90 s. The SiO_2_ was subsequently deposited using an e-beam evaporator (Korea Vacuum Tech, Inc.). After deposition of SiO_2,_ the remained PR part was then removed by Acetone via ultrasonication and NDA structure finally fabricated.

### Fabrication device

OLEDs and low-index NDA integrated OLED devices were fabricated using a thermal evaporator (Digital Optics Vacuum, Inc.). The organic layers and metal cathode were deposited under a low pressure of –10–6 Torr at deposition rates of 0.1 nm/s and 0.4 nm/s, respectively. Sixty nanometers of N,N′-di(1-naphthyl)-N,N′-diphenyl-4,4′-diamine (NPB) was used as a hole transport layer, 80 nm of tris(8-hydroxyquinolianto)-aluminum (Alq3) was used as an emission and electron transport layer, 0.8 nm of lithium fluoride (LiF) was used as an electron injection layer, and 200 nm of aluminum (Al) was used as a cathode. Before the fabrication of the OLEDs, the ITO substrate with low-index NDA was treated using UV–ozone (AHTECH LTS, Inc.) and O2–plasma (Femto Science Inc.), in that order.

### Measurement

The electroluminescence (EL) characteristics of the fabricated OLEDs with four discrete active emission area (6.25 mm × 6.25 mm) were measured with a spectroradiometer (PR-670, Photo Research, Inc.) and source meter (Keithley Model 237, TEKTRONIX Inc.) in a dark box. Detail calculations of current density is written in Supporting Information S4.

## Supplementary information

Supplementary information.
