# Supplementary material for: Enhanced optical efficiency and color purity for organic light-emitting diodes by finely optimizing parameters of nanoscale low-refractive index grid
Source: Sci Rep. 2020 Mar 27;10:5631. doi: 10.1038/s41598-020-62470-5 (PMC7101331; doi:10.1038/s41598-020-62470-5)
Supplement: Supplementary file 1 — Supplementary information. [file 41598_2020_62470_MOESM1_ESM.docx]

**Supporting Information**

Enhanced optical efficiency and color purity for organic light-emitting diodes by finely optimizing parameters of nanoscale low-refractive index grid

**Jae Geun Kim^1,§^, Yooji Hwang^1,§^, Ha Hwang^1^, Jun Hee Choi^1^, Young Wook Park^2,*^ and Byeong-Kwon Ju^1,*^**

^1^Display and Nanosystem Laboratory, School of Electrical Engineering, Korea University Seoul 136-713, Republic of Korea

^2^School of Mechanical and ICT Convergence Engineering, SUN MOON University, Chungcheongnam-do 31460, Republic of Korea

^*^Correspondence and requests for materials should be addressed to Y.W.P. (email: zerook@sunmoon.ac.kr) or B.-K.J. (email: bkju@korea.ac.kr)

Phone No.: +82-2-3290-3665

Fax. No.: +82-2-3290-3791

^§^These authors contributed equally to this work

1. ***FDTD simulation results according to the pitch, height and coverage of NDA***


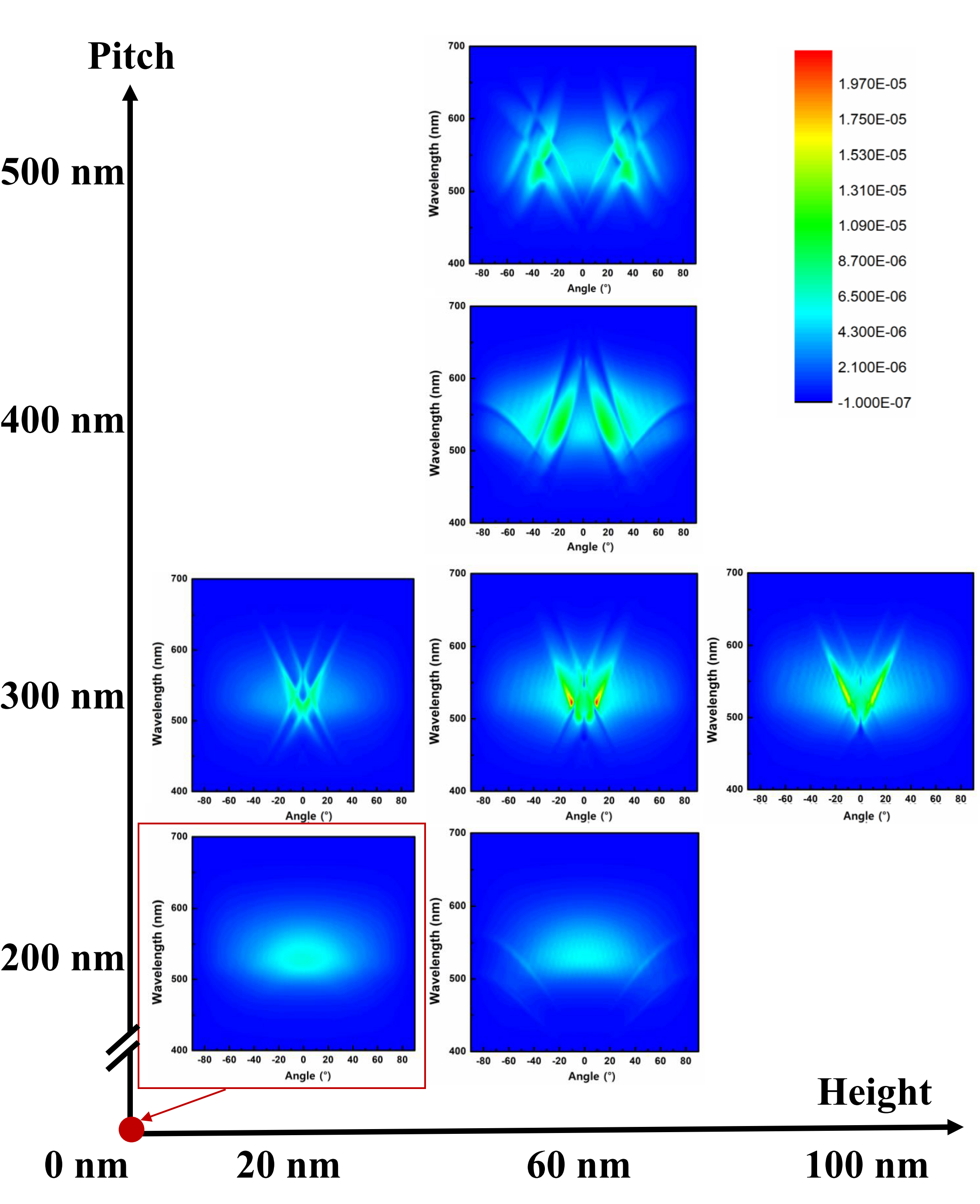


***Figure S.1. FDTD simulation results on luminous intensity according to viewing angle when height and pitch are changed. The contour graph in the red box is the reference result without NDA.***


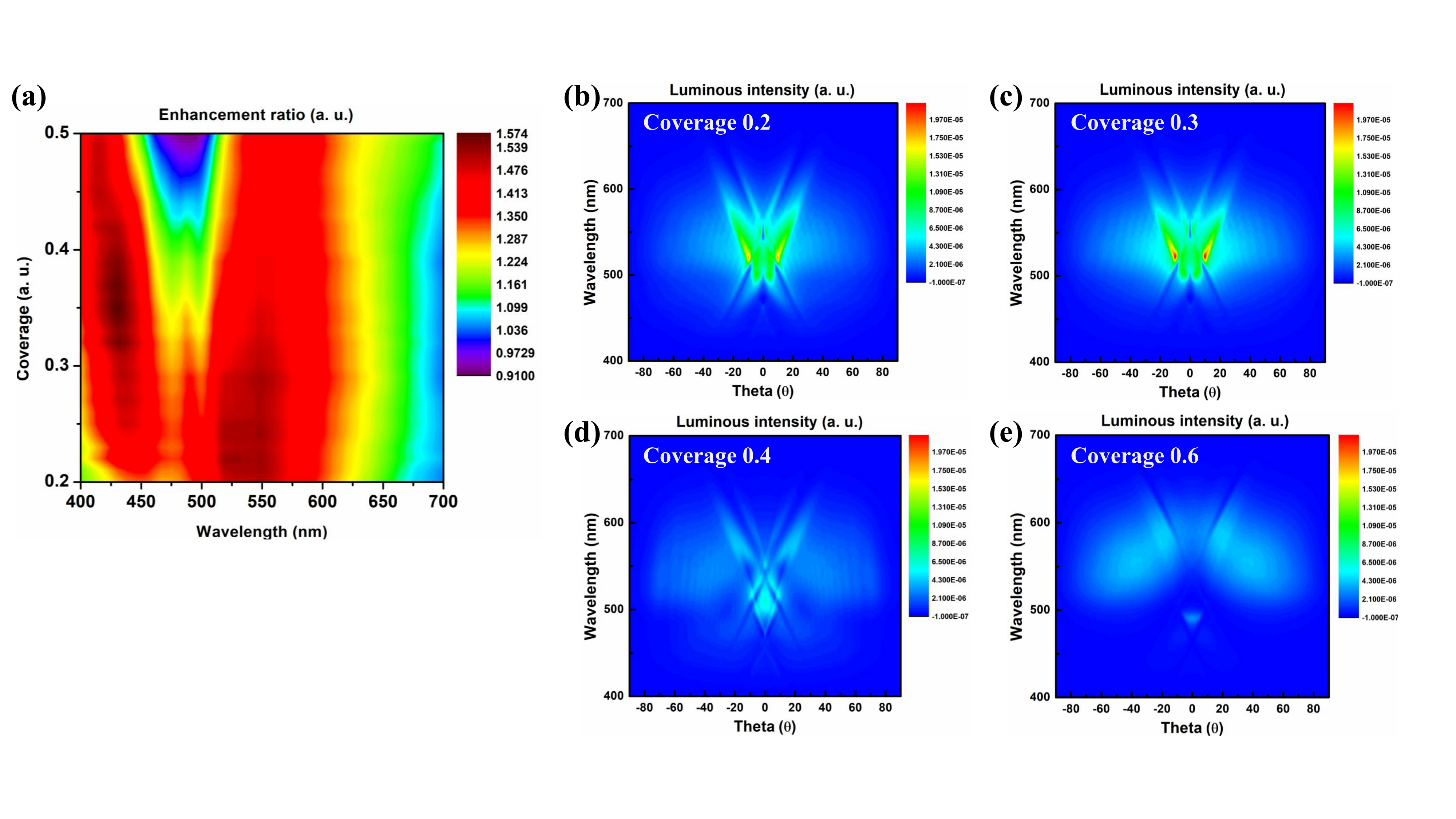


***Figure S.2. (a) Enhancement ratio and (b) luminous intensity according to coverage parameter of the NDA structure via 2-dimensional FDTD simulation results.***

***S.2. Fabricating hexagonal array of the NDA structure using 3-beam Lloyd’s mirror stage via LIL process.***


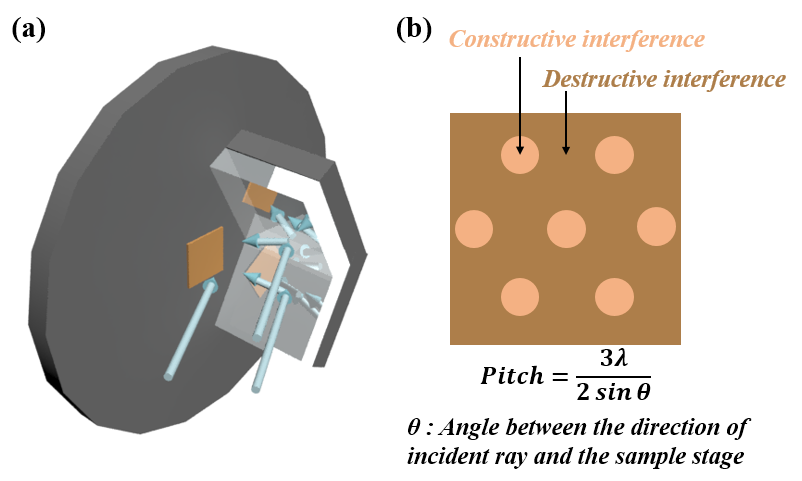


***Figure S.3. (a) Schematic diagram of fabricating hexagonal arrayed NDA structure using 3-beam Lloyd’s mirror. (b) Constructive-destructive interference on sample when the 3-beam Lloyd’s mirror is used for exposure during LIL process.***

***S.3. EL characteristics of fabricated OLED devices according to the height of NDA***


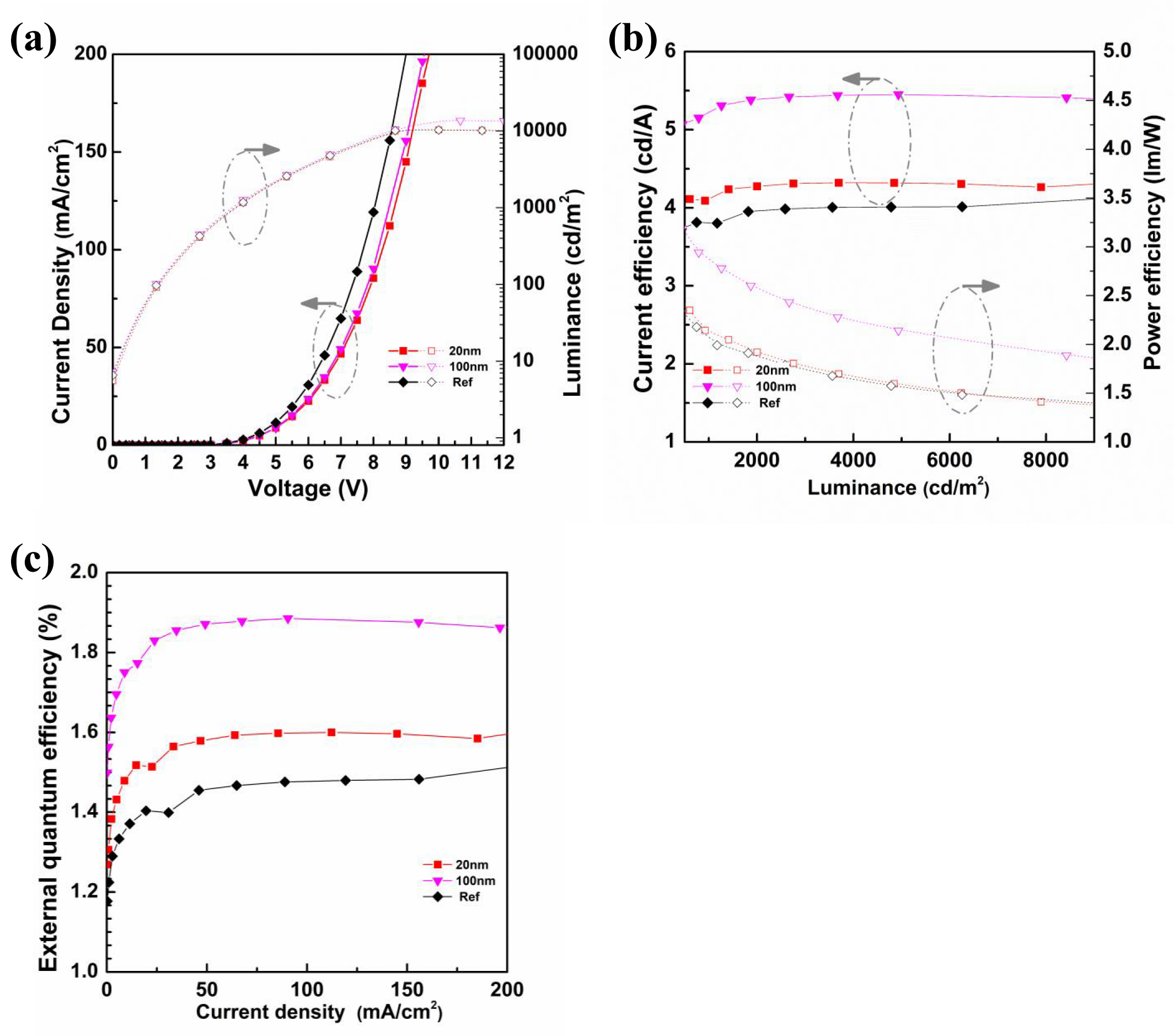


***Figure S.4. Characteristics of devices with rectangular dot array. (a) Current density and luminance per voltage, (b) current efficiency and power efficiency per luminance, (c) external quantum efficiency per current density, (d) electroluminescence results of each device and simulation.***

*OLED devices were fabricated for investigating the EL characteristics according to the height of the NDA, separately from the main manuscript. The Ref device is Glass / ITO (180 nm) / NPB (60 nm) / Alq_3_ (80 nm) / LiF (0.7 nm) / Al (100 nm). Device-1 and Device-2 have Glass / ITO (180 nm) / NDA / NPB (60 nm) / Alq_3_ (80 nm) / LiF (0.7 nm) / Al (100 nm) structures with NDA heights of 20 nm and 100 nm, respectively.*

*Figure S.4(a) shows J-V-L characteristics by height dependence. Leakage current, which could occur when the light extraction structure is inserted between the anode part and organic layer, has not occurred. While preserving characteristics of intrinsic diode current density, our devices show higher outcoupling effect as a finely optical effect. Figure S.4(b) shows current efficiency and power efficiency by luminance. In terms of current efficiency, Device-1 to Device-3 demonstrate enhanced results as the heights of the devices increase at 100 mA/cm^2^. Although the current densities of NDA devices were increased, current efficiencies were also enhanced because of light extraction. Consistently, in terms of power efficiency, NDA devices also exhibit higher power efficiencies in contrast to that of the Ref device. As a result, the EQEs of the NDA devices demonstrate enhanced results, as illustrated in Fig. S.4 (d). Detailed results about efficiencies are summarized in Table S.1.*

***Table S.1. Summary of rectangular array devices varying with NDA height characteristics***

|  | **Current efficiency**  **[cd/A]** | **Power efficiency**  **[lm/W]** | **External quantum efficiency**  **[%]** |
| --- | --- | --- | --- |
| **Device-1** | 4.32 (+ 7.86%) | 1.70 (+ 1.12%) | 1.60 (+ 8.25%) |
| **Device-2** | 5.45 (+ 36.12%) | 2.14 (+27.61%) | 1.89 (+ 27.75%) |
| **Ref** | 4.01 | 1.68 | 1.48 |

***S.4. Calculations of current density of NDA-integrated OLED devices***

*Since the NDA structure is composed of insulating material (SiO_2_), defining effective cell area crucially leads to calculations of optical efficiencies of fabricated OLED devices. If the measured current density and luminance is denoted as J and L respectively, the effective current density and luminance from defined cell area can be represented as following where the effective aperture ratio is r %.*

*Current density of nano-emissive effective area = J/r*

*Luminance of nano-emissive effective area = L/r*

*External measured current density = J*

*External measured luminance = L*

*Current efficiency =* $\frac{L}{J}(Measured)=\frac{\left( L/r \right)}{\left( J/r \right)}(Effective defined cell area)$

*From above perspective, the optical efficiency measurements of the entire OLED device area are valid because the luminance and current density are divided by aperture ratio (r) at the same time.*
